# Neurochemistry of major depression: a study using magnetic resonance spectroscopy

**DOI:** 10.1007/s00213-014-3687-y

**Published:** 2014-07-31

**Authors:** Beata R. Godlewska, Jamie Near, Philip J. Cowen

**Affiliations:** 1 University Department of Psychiatry, Warneford Hospital Oxford, Oxford, OX3 7JX UK; 2 FMRIB Centre, Department of Clinical Neurology, University of Oxford, Oxford, UK

**Keywords:** Depression, GABA, Glutamate, Glutathione, SSRIs, Magnetic resonance spectroscopy (MRS)

## Abstract

**Rationale:**

Magnetic resonance spectroscopy (MRS) is an acceptable non-invasive means of studying brain neurochemistry in depression. Previous studies in depressed patients have focused on measurement of the amino acid neurotransmitters, γ-aminobutyric acid (GABA) and glutamate.

**Objectives:**

The aim of this study is to use MRS in conjunction with the ultrashort echo time ‘SPECIAL’ technique to measure cortical levels of GABA, glutamate and glutathione (GSH) levels in unmedicated patients with major depression. We also examined the effect of 6-week treatment with the selective serotonin re-uptake inhibitor, escitalopram.

**Methods:**

We studied patients with DSM-IV major depression and healthy age-matched controls using proton MRS. GABA, glutamate and GSH were measured relative to creatine in a voxel placed in occipital cortex.

**Results:**

There was no difference in GABA or glutamate levels between depressed participants and controls; however, depressed patients had lower GSH levels. Six-week escitalopram treatment, which resulted in significant clinical responses in some patients, did not alter concentrations of GABA, glutamate or GSH.

**Conclusions:**

The sources of variability of GABA and glutamate measures in different studies of depressed patients require further study. Our results suggest that concomitant treatment with selective serotonin re-uptake inhibitors (SSRIs) is unlikely to be an important confounding factor. If lowered GSH levels can be confirmed, they may represent the presence of oxidative stress in some depressed patients.

## Introduction

Magnetic resonance spectroscopy (MRS) provides an acceptable and non-invasive means of assessing aspects of brain neurochemistry in depressed patients in vivo. Particular attention has focused on levels of the amino acid neurotransmitters, glutamate and γ-aminobutyric acid (GABA) because of their postulated role in the pathophysiology of depression (Taylor et al. 2003; Sanacora et al. 2008; Sanacora 2010). Findings to date in MRS studies in patients with major depression have not been particularly consistent, probably related to methodological factors and likely patient heterogeneity. However, a recent meta-analysis of studies of glutamate in depressed patients indicated that MRS glutamate was likely to be decreased in acute depression, particularly in anterior brain regions (Yüksel and Öngür 2010). GABA concentrations are also reported to be lowered in depression, particularly in occipital cortex where GABA is easier to measure with current MRS methodology (Sanacora et al. 1999, 2004; Hasler et al. 2007).

Glutathione (GSH) is a major endogenous free radical scavenger, and its reduction can increase vulnerability to cellular oxidative stress (Berk et al. 2008). There is evidence that patients with depression have decreased antioxidant capacity in plasma as shown by lowered levels of glutathione peroxidase (GPX) (Maes et al. 2011). Also a post-mortem study of prefrontal cortex in brain tissue, derived from the Stanley Foundation Consortium, found lowered levels of both GSH and GPX in patients diagnosed with major depression (Gawryluk et al. 2011). A recent MRS study reported lower levels of GSH in occipital cortex in both patients with major depression and those with chronic fatigue syndrome (Shungu et al. 2012).

In conjunction with proton MRS, the short echo time ‘SPECIAL’ technique (Mekle et al. 2009; Near et al. 2013) enables simultaneous detection of GSH as well as several other neurometabolites, including GABA, glutamate and glutamine in a single acquisition, without the need for spectral editing. The primary aim of this study was to compare GABA, glutamate, glutamine and GSH levels in occipital cortex (OCC), in unmedicated patients with major depression and healthy controls. The secondary aim was to assess the effects of 6-week treatment with the selective serotonin re-uptake inhibitor (SSRI), escitalopram, on glutamate, glutamine, GABA and GSH. This investigation was part of a combined study that also looked at the ability of escitalopram to change the neural substrate of emotional processing measured by functional magnetic resonance imaging (fMRI). Results from this part of the study will be reported separately.

## Methods

### Participants, mood ratings and antidepressant treatment

Participants were recruited through mental health clinics and advertising in local newspapers. Before enrolment to the study volunteers were assessed for current and past Diagnostic and Statistical Manual of Mental Disorders, Fourth Edition Text Revision (DSM-IV)-TR depressive disorder and other Axis 1 diagnoses by the Structured Clinical Interview for DSM-IV Axis I Disorders Schedule (SCID-I) (First et al. 1997).

The final participant sample included 39 patients with major depression and 31 healthy controls with a similar age and gender distribution. Exclusion criteria from the study were as follows: for depressed patients—suffering from psychosis or substance dependence as defined by DSM-IV, being at clinically significant risk of suicidal behaviour, having contraindications to escitalopram treatment or being treated with psychiatric medication less than 3 weeks before the beginning of the study; for healthy volunteers—current or past history of Axis I disorder as defined by DSM-IV; and for both groups —major somatic or neurological disorders, pregnancy or breast-feeding, contraindications to MRS imaging. The study was approved by Oxford Research Ethics Committee in accordance with the Helsinki Declaration of 1964. All participants gave written informed consent prior to their inclusion in the study. Participants were reimbursed for their time.

Mood ratings were measured using the Hamilton Rating Scale for depression (HAM-D) (Hamilton 1960) and the Beck Depression Inventory (BDI) (Beck et al. 1961), while anxiety ratings were scored using the Spielberger’s State-Trait Anxiety Inventory (Spielberger et al. 1993). Ratings were carried out immediately prior to MRS scanning. After the first MRS scan, depressed patients were treated for 6 weeks with escitalopram, 10 mg daily, following which MRS scanning was carried out again.

### MRS methodology

Scanning was performed on a 3T Siemens TIM Trio scanner (Erlangen, Germany) with a body coil transmitter and a 32-channel receive head array. Data were acquired from a 20 × 25 × 20 mm voxel located in the occipital cortex (OCC). The voxel was positioned manually by reference to an axial T1-weighted gradient echo image.

SPECIAL data with water suppression were acquired (TE 8.5 s, 3,200 ms, 16-step phase cycle, 128 averages for the occipital voxel). Six outer volume suppression slabs were applied (one on each side of the cubic voxel) to suppress signals originating from outside the volume of interest and to minimise motion-related image-selected in vivo spectroscopy (ISIS) subtraction artefacts. For all spectra, a semi-automated processing chain was applied, which involved the removal of motion-corrupted averages and frequency and phase drift corrections prior to signal averaging. Motion-corrupted averages were always discarded in combination with the corresponding ISIS subtraction pair; however, to prevent signal-to-noise ratio (SNR) loss, the remainder of the 16-step phase encode cycle was not discarded. Following spectral processing, SPECIAL data were analysed with LCModel version 6.2-2B (Provencher 1993) using a basis set that consisted of 21 simulated metabolite basis spectra. Basis spectra were generated using an in-house, MATLAB (Natick MA, USA) based implementation of the density matrix formalism to simulate the effect of the SPECIAL pulse sequence on each of the 21 metabolite spin systems studied. All metabolite concentrations were calculated in reference to total creatine (creatine + phosphocreatine). Quantification reliability and spectral quality were assessed using Cramer-Rao lower bounds (CRLB), spectral linewidth and SNR, and strict quality limits were enforced. Specifically, concentration estimates with CRLB >20 % were rejected as unreliable and all spectra with linewidth greater than 8 Hz (one spectrum) or SNR less than 50 (one spectrum) were removed from further analysis. SNR was defined as the maximum (real) signal intensity between 0.2 and 4.2 ppm divided by the standard deviation of the fit residual over the same frequency range. Linewidth was defined as the full width at half maximum of the fitted peaks. T1-weighted structural images were of whole brain and were acquired with 1-mm^3^ voxel resolution, and FSL FAST was used to segment the structural brain images into grey matter, white matter and CSF, to allow estimation of voxel composition (for more information about the accuracy and reliability of SPECIAL in conjunction with the LCModel in the measurement of GABA concentrations, see Near et al. (2013)).

Group differences were analysed using unpaired *t* tests or MANOVA in SPPS v.20 (SPSS Inc., Chicago, IL, USA). Effects of escitalopram treatment were compared by paired *t* tests. Correlations were carried out using Pearson’s product moment.

## Results

### Participant characteristics

There was no significant difference in age and gender ratio between patients and controls. The ratings on the HAM-D score showed that the majority of the patients were moderately depressed. Ten met DSM-IV criteria for major depression with melancholia; three patients had a history of panic attacks; and one had comorbid generalised anxiety disorder. The mean antidepressant free period for patients with recurrent depression was 111 weeks (range 12–468); however, 22 patients had never received treatment with antidepressants. Two patients were treatment-resistant during the current episode, each having received three courses of antidepressants (one, two courses of SSRIs and one course of mirtazapine and the other, two courses of SSRIs and one course of lofepramine). Demographic data are presented in Table 1.

**Table 1:** Demographic data for patient and control groups

	Patients (*n* = 33)	Controls (*n* = 27)
Gender, F/M	19:14	16:11
Age (years)	29.9 ± 10.6	30.3 ± 10.6
HAM-D	22.3 ± 4.6	0.3 ± 0.7
BDI	30.1 ± 6.4	0.7 ± 1.3
STAI-T	61.9 ± 8.00	28.8 ± 7.7
STAI-S	49.1 ± 10.7	27.1 ± 6.6

Data presented as mean ± SD, and numbers of participants

*HAM-D* Hamilton Rating Scale for Depression, *BDI* Beck Depression Inventory, *STAI-T* and *STAI-S* Spielberger’s State-Trait Anxiety Inventory

### MRS analysis

**Fig. 1:**
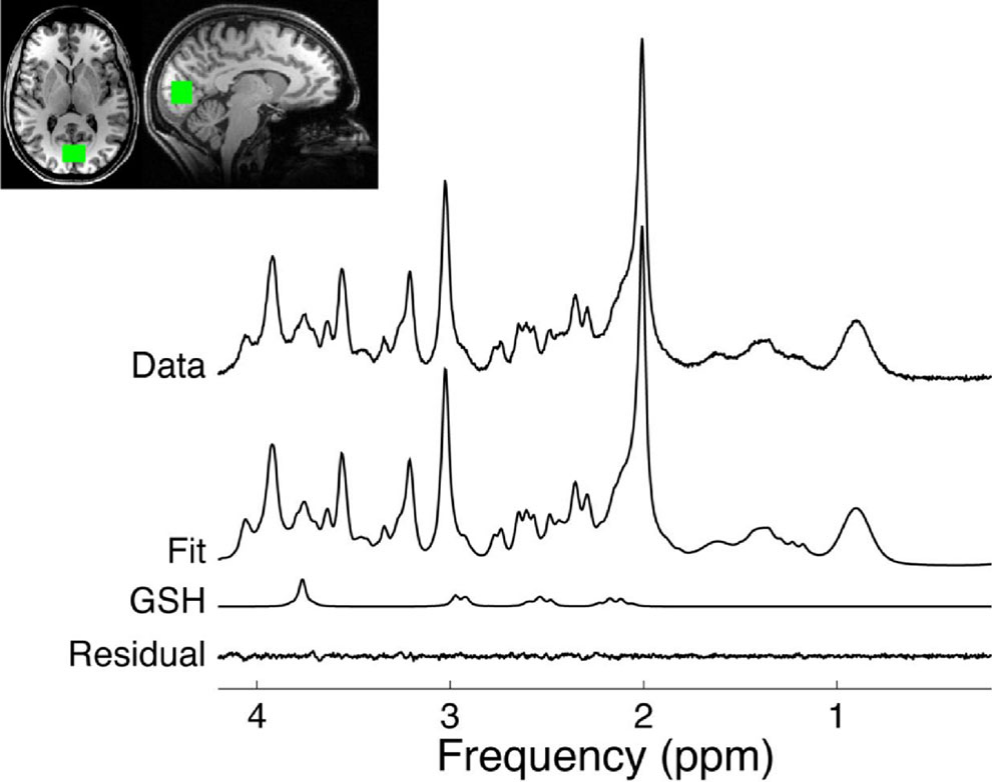
Representative spectrum from the occipital cortex (OCC) along with the LCModel fit and residual. The location of the region of interest is also shown

Representative spectra and the corresponding voxel placement are shown in Fig. 1 Also shown are the spectral fits as well as the residuals (data fit), where the flatness of the residuals can serve as an indicator of the goodness of fit. Due to technical limitations (see above) we excluded six patients and four healthy controls from the between-group analysis. Within-subject analysis for the effect of escitalopram treatment was carried out in 27 patients for whom valid data from baseline and 6-week scans were available.

The two groups did not differ significantly in terms of voxel composition in regards to white matter and cerebrospinal fluid content; however, grey matter content was significantly lower in the patient group (Table 1), and grey matter content was therefore entered as a covariate into the comparison of depressed patients and controls. There were no significant differences in any measures of voxel content when the pre-treatment and treatment scans of the depressed patients were compared (data not shown).

**Fig. 2:**
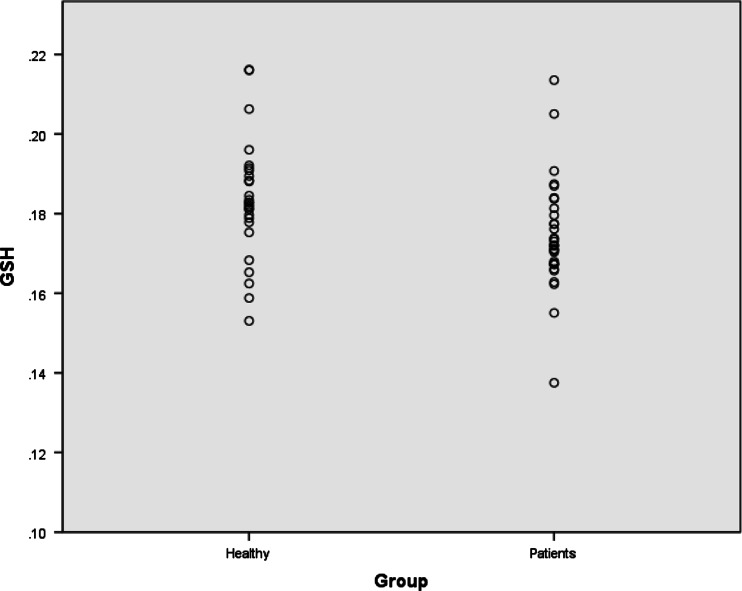
Individual GSH concentrations referenced to creatine in occipital cortex in depressed patients and healthy controls

The MANOVA showed that depressed patients had lower GSH levels (*F* = 5.10, *p* = 0.028) (Fig. 1), but depressed patients and controls did not differ in concentration of any other metabolites (GABA, glutamine, glutamate; all *p* values, *p* > 0.3) (Fig. 2, Table 2). Adding age and gender to the MANOVA lowered the statistical significance of the GSH reduction (*F* = 4.28; *p* = 0.042) and did not alter findings with any of the other metabolites. There was no correlation between clinical ratings and any of the metabolite levels (all *p* values, *p* > 0.5), and examination of the small number of melancholic depressed patients separately also showed no difference from controls (all *p* values, *p* > 0.1). Six-week escitalopram treatment, which resulted in clinical responses (50 % or greater decrease in HAM-D) in 18 patients, did not alter concentrations of GABA, glutamate, glutamine or GSH (Table 3). Similarly, there was no correlation between change in HAM-D and change in any metabolite level (all *p* values, *p* > 0.1).

**Table 2:** Comparisons of the voxel content and magnetic resonance spectroscopy (MRS) measures relative to total creatine (mean ± SD) for glutathione, GABA, glutamate and glutamine, across both patients and controls, corrected for grey matter content

	Patients	Healthy	Significance value
Grey matter	0.38 ± 00.072	0.421 ± 0.070	(*t*(28) = 2.191, *p* = 0.033
White matter	0.510 ± 0.056	0.490 ± 0.053	(*t*(28) = −1.422, *p* = 0.161
CSF	0.109 ± 0.047	0.089 ± 0.032	(*t*(28) = −1.912, *p* = 0.061
GSH	0.174 ± 0.013	0.183 ± 0.014	*F* = 5.098, *p* = 0.028
GABA	0.225 ± 0.034	0.237 ± 0.053	*F* = 0.507, *p* = 0.33
Glutamate	0.890 ± 0.085	0.880 ± 0.090	*F* = 0.001, *p* = 0.97
Glutamine	0.192 ± 0.036	0.187 ± 0.030	*F* = 0.008, *p* = 0.93

**Table 3:** Magnetic resonance spectroscopy (MRS) measures relative to total creatine (mean ± SD) for glutathione, GABA, glutamate and glutamine before and after 6 weeks of treatment with 10 mg escitalopram (*n* = 27)

	Before treatment	After 6 weeks of treatment	*p* value (paired *t* test)
GSH	0.174 ± 0.012	0.177 ± 0.014	0.28
GABA	0.226 ± 0.032	0.221 ± 0.035	0.52
Glutamate	0.893 ± 0.086	0.865 ± 0.088	0.08
Glutamine	0.188 ± 0.036	0.188 ± 0.039	0.99

## Discussion

The principal finding of our study is that, compared to controls, depressed patients had lower levels of GSH in occipital cortex. No significant change was seen in the other neurometabolites measured, that is, GABA, glutamate and its precursor and metabolite glutamine. Further, we found that 6-week treatment with the SSRI, escitalopram, had no effect on any of the metabolite levels, despite many of the patients experiencing clinically significant improvements in depressive symptomatology.

GSH is an important antioxidant in the CNS, and GSH depletion is believed to contribute to various forms of cell death (Bains and Shaw 1997). GSH has been relatively little studied in MRS studies of depressed patients; nevertheless, as noted in the “Introduction” section, peripheral measures of antioxidant capacity in depression have shown reductions in GPX, an enzyme whose role is to help protect the organism from oxidative damage (Maes et al. 2011). A post-mortem study in depressed patients also showed lowered levels of GPX and GSH in cortex (Gawryluk et al. 2011). In agreement with our findings, one previous MRS study found lowered cortical GSH in relative to healthy controls in both patients with major depression and patients with chronic fatigue syndrome (Shungu et al. 2012). While the latter study also employed a voxel in occipital cortex, spectral acquisition was carried out with point-resolved spectroscopy and J editing (Shungu et al. 2012). However, two recent MRS studies in bipolar disorder failed to find differences in cortical GSH between bipolar patients and controls (Godlewska et al. 2014; Lagopoulos et al. 2013) suggesting that lowered GSH is probably not a feature of mood disorders in general.

There is increasing interest in the role of glutamateric mechanisms in depression, and MRS has been widely used to measure glutamate in patients with mood disorder (Sanacora et al. 2008). At conventional MR field strengths, it is technically difficult to separate glutamate from glutamine, and the two are often reported together as a composite measure called Glx. SPECIAL, however, does allow the differential resolution of glutamate and glutamine which were therefore measured separately in the current study (Mekle et al. 2009). However, no change between depressed patients and controls was found in either metabolite or when the two were summed together (data not shown).

The majority of MRS studies of Glx in depressed patients have found lowered levels in anterior brain regions, though the data are not completely consistent (Yüksel and Öngür 2010; Price et al. 2009; Taylor et al. 2012). There is a piece of evidence that lowered Glx may be more apparent in patients with chronic depression (Portella et al. 2011), so altered glutamate activity might be restricted to a subgroup with more sustained and severe illness. Glx has been less studied in occipital cortex, and one study in acutely depressed patients reported an increase in glutamate (Sanacora et al. 2004); we also found increased Glx in occipital cortex in recovered depressed patients (Bhagwagar et al. 2007) and increased glutamate in the same brain region in euthymic young people at risk of depression through having a depressed parent (Taylor et al. 2011). More recently, we have also found elevated levels of Glx in the hippocampus in young people at increased risk of depression (Mannie et al. 2014).

Our current finding of unaltered Glx in occipital cortex in acute depression is in agreement with a recent study that found no difference in occipital Glx between depressed patients, patients with chronic fatigue and controls (Murrough et al. 2010), as well as an earlier investigation that included both medication-naive and medication-resistant depressed patients (Price et al. 2009). These disparate findings make it hard to form a coherent picture of MRS glutamate abnormalities in depressed patients. It is possible that stage of illness might be important with ‘at-risk’ individuals showing increased Glx levels followed by a decline over the course of recurrent episodes of depression (de Diego-Adeliño et al. 2013). Changes may also depend on the brain region studied (Taylor et al. 2010).

GABA concentration has also been reported to be lower in MRS studies in depression, in both anterior brain regions and in occipital cortex, though again not all studies are in agreement (Sanacora et al. 1999, 2004; Hasler et al. 2007; Walter et al. 2009; Murrough et al. 2010). A previous investigation in a large group of unmedicated depressed patients found that levels of GABA in occipital cortex were markedly lower than controls in patients with melancholic depression but reduced to lesser extent in those with non-melancholic forms of illness (Sanacora et al. 2004). Thus, severity, or the particular symptom profile associated with melancholic depression, might be more clearly associated with lowered GABA levels as measured by MRS. Perhaps relevant to this is the study of Price et al. (2009) which found diminished GABA levels in both anterior cingulate cortex and occipital cortex in depressed patients who had been refractory to at least three courses of antidepressant treatment during their current episode of illness. However, other depressed patients, either treatment-naive or with lesser degrees of treatment resistance did not show lower GABA levels in either brain region. We therefore also analysed our GABA data by comparing the GABA levels of patients who failed to show a clinical response to escitalopram treatment over the next 6 weeks with those who showed at least a 50 % decrease in HAM-D but found no difference in pre-treatment (baseline) GABA levels between responders and non-responders (data not shown). However, the treatment resistance of these patients was clearly less than those studied by Price et al. (2009).

We found no significant effect of 6-week treatment with escitalopram on any of the neurometabolites that we studied. There is little work on the effects of sub-chronic antidepressant treatment on Glx concentrations in MR studies of depressed patients, though our findings are in agreement with previous work from our group that studied the effect of 1-week treatment with escitalopram in depressed patients (Taylor et al. 2012). There is, however, a previous report that SSRIs increase occipital GABA levels in depressed patients over about 2 months of treatment (Sanacora et al. 2002), and a similar effect was reported with electroconvulsive therapy (Sanacora et al. 2003). In the latter studies, however, GABA levels were lowered relative to controls prior to the treatment interventions, perhaps making an increase easier to demonstrate.

We used a novel form of spectral acquisition (SPECIAL) which employed short echo times to allow identification of several neurometabolites without the need for spectral editing (Mekle et al. 2009). It is possible therefore that the lack of differences we have found between depressed patients and controls in concentrations of glutamate and GABA could be attributable to the particular MRS technique that we applied. For example, edited MRS measures of GABA are known to contain contaminating signals from macromolecules, whereas the macromolecule contribution to short echo time GABA measures is not well known. Nonetheless, we have previously shown that there is a highly significant correlation between occipital GABA levels measured with SPECIAL and a more conventional spectral editing technique (Near et al. 2013). Unexpectedly, we found that compared to controls, the depressed patients had a lower grey matter content in the occipital voxel selected for MRS study. While some investigations have found decreases in grey matter in certain brain regions in depression, the occipital cortex is not usually implicated (Kupfer et al. 2012). In fact, in the present study, we have also compared overall brain structure between the depressed patients and controls using voxel-based morphometry and found no significant differences (data not shown). We therefore believe that the decrease in grey matter in the occipital voxel in the depressed patients is likely to be a chance finding.

Another potential limitation of our study is the use of creatine as a reference because it is possible that certain psychiatric disorders might be associated with changes in brain creatine levels (Öngür et al. 2009). We think this is unlikely because an abnormality in creatine levels would be expected to lead to more widespread differences in metabolite ratios between patients and controls; however, it would have been preferable to use a method capable of measuring absolute concentrations of the metabolites of interest. Finally, the use of occipital cortex may have limited relevance to understanding biochemistry of depression because functional imaging in depression typically shows changes in anterior brain regions involved in emotional regulation (Kupfer et al. 2012).

In conclusion, our studies indicate that GSH levels may be decreased in patients with major depression, supporting a previous MRS investigation in depression as well as theoretical approaches that implicate impaired antioxidant mechanisms in the pathophysiology of mood disorder. However, in our patient group, we did not find evidence of decreases in cortical Glx and GABA as has been reported by many other studies. It is important to understand the reason for the discrepant MRS findings in depressed patients, and our study suggests that concomitant treatment with SSRIs may not be an important confounding factor. It seems more likely that patient heterogeneity plays an important role, and in this context, it seems worth noting that our patients, in general, were not severely depressed, nor treatment resistant. In future work, it will be important to identify the clinical characteristics that are most reliably associated with alterations in cortical Glx and GABA.
